# Assessment of Prescriptions in Elderly Patients Hospitalized in Medicine Departments

**DOI:** 10.3390/jcm10225343

**Published:** 2021-11-16

**Authors:** Audrey Giroux, Christelle Prudent, Pierre Jouanny, Géraldine Muller, Hervé Devilliers, Lucie Vadot

**Affiliations:** 1 Pharmacy Department, Dijon University Hospital Center, 21000 Dijon, France; christelle.prudent@chu-dijon.fr (C.P.); lucie.vadot@chu-dijon.fr (L.V.); 2 Geriatric Internal Medicine Department, Dijon University Hospital Center, 21000 Dijon, France; pierre.jouanny@chu-dijon.fr; 3 Internal Medicine Department, Dijon University Hospital Center, 21000 Dijon, France; geraldine.muller@chu-dijon.fr (G.M.); herve.devilliers@chu-dijon.fr (H.D.)

**Keywords:** aged, potentially inappropriate medication list, clinical audit, geriatrics, inappropriate prescription, misuse, overuse

## Abstract

Drug-related iatrogenesis is an important issue in the elderly population, and preventing iatrogenic accidents helps to reduce hospitalizations. Our study’s objective was to evaluate prescriptions in the geriatric population of our establishment. The study conducted is a targeted clinical audit. Ten criteria were tested on the hospital prescriptions of people over 75 years old in 11 medical departments, before and after improvement actions. The non-compliance threshold was set at 10% of prescriptions for each criterion. In each phase, 165 patients were included. Four criteria were non-compliant (NC) in the first phase: the presence of Potentially Inappropriate Medications for the Elderly (PIMs) (NC = 57.6%), the adaptation of the medication to renal clearance (NC = 24.9%), the presence of illogical combination (NC = 9.7%), and the total anti-cholinergic score of the prescription (NC = 12.1%). After the implementation of improvement actions, the number of non-compliant criteria decreased between the two phases, from four to two. We obtained a significant improvement for three of the four criteria found to be non-compliant in the first phase. The criterion adaptation to renal function is close to compliance (NC = 10.1%) and the PIMs criterion remained non-compliant after reassessment (NC = 32.1%). Vigilance must be ongoing in order to limit drug iatrogeny, particularly in frail elderly patients.

## 1. Introduction

On 1 January 2021, 20.7% of the French population was older than 65 years [1]. Several factors explain this increasing aging of the population, which will likely continue in the years to come. Aging is a very heterogeneous phenomenon depending on the subject [2], and optimal and preventative medical care can help a patient achieve successful aging without altering their functional reserves [3]. Furthermore, drug-related iatrogenesis is more important in the elderly population, not only because of higher rates of polypharmacy, but also because of pharmacokinetic and pharmacodynamic changes in the aging body. Indeed, iatrogenic accidents are two to three times more frequent after the age of 65 [4]; serious adverse events (SAEs) related to care represent 10% of hospitalizations for elderly subjects, and this rate rises to 20% for subjects over 80 years of age [5]. Thirty-nine percent of these SAEs are due to medication, and it is estimated that almost two-thirds are preventable iatrogenic events [5,6]. Preventing these events helps to limit potential alterations in an elderly subject’s condition and reduces hospitalizations [6]. Decreasing drug iatrogenesis involves, among other things, re-evaluating treatments and better prescription practices (“le mieux prescrire” in French), i.e., avoiding, as much as possible, prescribing potentially inappropriate medications to the elderly.

At our university hospital center, a group of medical professionals ensures the safety of the medication circuit for the elderly and develops and implements propositions to improve it. The group Medication Management of the Elderly Patient (MMEP) has identified drug prescription as a risk in the elderly patient’s hospital pathway. In order to specify the risk and adapt management, we decided to conduct an assessment of good prescription practices for elderly patients in medical services. The aims of this study were: to describe the frequency of non-compliant prescriptions in a geriatric population hospitalized in our center, compared to a framework of good practices based on data from the literature (first phase); to propose and implement improvement actions; and to compare non-compliance rate between the baseline (first phase) and following the impact of improvement actions (second phase).

## 2. Materials and Methods

The study was observational with retrospective data collection. Ethics committee approval was not required, as confirmed by the local ethical board. Non-opposition was obtained from all participants in accordance with French law. Data were extracted from medical records according to inclusion criteria and treated anonymously.

For each of the two phases, every consecutive patient over 75 years of age who was hospitalized for at least 72 h from 1 January to 31 March 2018 (first phase) and from 1 June to 31 August 2018 (second phase) in one of the targeted services was eligible. Fifteen patients were included for each targeted service. Patients who died or were transferred to another department within the first 72 h were excluded. For the second phase, patients included in the first phase were not eligible.

The services included were the 11 medical departments with the highest number of elderly people in our hospital center: two departments of neurology, two of cardiology, two of internal medicine, two of geriatric internal medicine, as well as the endocrinology, hepato-gastroenterology, and rheumatology departments.

The patient characteristics collected were: age, sex, weight, BMI, blood albumin level, renal clearance according to the Cockroft–Gault formula, length of hospitalization, number of drugs prescribed, place of origin, and dependence (for at least one daily life activity).

The study was conducted as a Targeted Clinical Audit (TCA).

The TCA was conducted in two phases: a first phase from January 2018 to March 2018, consisting of the evaluation of prescriptions, followed by the implementation of improvement actions, and a second phase from June to August, during which the impact of the improvement actions was assessed. Phase 2 was to begin one month after the presentation of the improvement actions to the healthcare professionals at the end of phase 1.

The evaluation focused on the prescriptions of the last 24 h of hospitalization, based on three frames of reference [7,8,9,10,11].

The first one is the evaluation of professional practice (Evaluation des Pratiques Professionnelles, EPP) titled *Prescription medication in the very elderly* and developed by the Collège Professionnel des Gériatres Français (CPGF) and the Haute Autorité de Santé (HAS) [11]. We used the website *Prescription guidelines for renal adaptation* (Guide de Prescription et Rein), developed by the French Society of Nephrology for the criterion of adaptation to renal function. The second frame of reference is a list entitled *Potentially Inappropriate Medications for the Elderly* (PIMs), collated by us from the literature referring to the elderly, namely the Laroche [7] and Beers [8] lists, and The Screening Tool of Older Persons’ Prescriptions (STOPP) criteria from the O’Mahony list [9]. These different lists categorize the drugs to be preferred or avoided according to the clinical situations, co-morbidities, and co-prescriptions of each elderly patient. The Laroche list is an adaptation of the Beers list to French practice. The PIMs list thus constituted contains 54 criteria divided into seven categories. Finally, we used the Anti-cholinergic Impregnation Coefficient (AIC) to define the anticholinergic potential of the prescriptions [10]. This scale assigns a score from 1 to 3 to each drug with anticholinergic properties according to its anticholinergic potential (weak = 1, strong = 3). A total of 10 items were selected in order to evaluate good prescribing practices in the elderly (Table 1). Each of the 10 criteria were evaluated during the two phases of the audit. The main endpoint was the rate of non-compliance with each of the criteria. The non-compliance threshold, defining the need to propose improvement actions, was set at 10%.

The descriptive analysis presents the quantitative variables in means and standard deviations (SD), and the qualitative variables are expressed by frequency.

The clinical characteristics of the two samples were compared using Student’s *t*-tests or chi-squared tests, depending on the variables studied. The significance threshold was set at 5% (*p* < 0.05) for all statistical tests.

We then compared the rate of non-compliance for each of the criteria between the two periods using chi-squared tests.

The analyses were performed using Epi-info 7^®^ version 7.2.2.6.

## 3. Results

### 3.1. First Audit Phase

#### 3.1.1. Patient Characteristics

During this phase, the prescriptions of 165 patients meeting the inclusion criteria were analyzed. The sex ratio was 1.04 and mean age (±SD) was 84.2 years (±5.3). The mean length of hospitalization was 9.9 days (±7.0), and patients had an average of 9.5 prescriptions (±3.4) on the day of collection.

#### 3.1.2. Compliant Criteria (Non-Compliance Rate < 10%)

Six criteria were compliant: the combination of more than two psychotropic drugs, the prescription of NSAIDs, the presence of more than one benzodiazepine, the prescription of cerebral vasodilators, drugs whose association is contraindicated, and the adaptation of the galenic formula to the patient’s abilities. (Table 2).

#### 3.1.3. Criteria Considered Non-Compliant (Non-Compliance Rate > 10%)

Over 10% non-compliance was observed for four criteria: PIMs (57.6% of non-compliance), non-adaptation to renal clearance (24.9%), anti-cholinergic score (12.1%), and illogical associations (9.7%) (Table 3).

A total of 133 PIMs were prescribed in 95 patients, with an average of 0.8 PIM/patient in the study. The most common PIMs were proton pump inhibitors (36.8%; *n* = 49) (inappropriate dosage or no indication), psycholeptics and benzodiazepines with a particularly long half-life (5.3%; *n* = 7), hydroxyzine (8.3%; *n* = 11), and central antihypertensive drugs (7.5%; *n* = 10).

The rate of prescriptions containing at least one drug not adapted to kidney function was 24.9% (*n* = 41). The therapeutic classes most concerned were analgesics (41.7%; *n* = 20) and oral antidiabetic drugs (22.9%; *n* = 11).

The illogical associations found were mostly class redundancies (50.0%; *n* = 8) or the combination of more than three antihypertensive drugs (37.5%; *n* = 6).

An overall anticholinergic score greater than 3 was mostly related to the prescription of several anticholinergic molecules and the prescription of hydroxyzine (12.1%; *n* = 20).

### 3.2. Improvement Approaches

Improvement actions targeting the four non-compliant criteria (criteria 6, 8, 9, and 10) were proposed and implemented. Our intervention was based on the training of prescribers, but also on pharmacists performing the daily pharmaceutical analysis of prescriptions. For this purpose, information sheets were made available to everyone, information meetings were held, and a method of targeting pharmaceutical analysis specific to elderly patients was developed.

First, prescribing aids were developed and made available to prescribers and pharmacists. Fact sheets were developed for the most commonly found PIMs: long half-life benzodiazepines, proton pump inhibitors (PPIs), alpha-blocking antihypertensive drugs, and hydroxyzine. A tool to help with total anticholinergic score calculation, as well as a fact sheet on the adaptation of therapeutics to renal function, were also prepared. All of these documents have been validated by the MMEP group as well as the *Potentially Inappropriate Medications for the Elderly* guidelines and were distributed to pharmacists and prescribers of the services covered by the audit.

In order to better target high-risk prescriptions, a new method of pharmaceutical analysis of prescriptions was implemented. Through daily queries, we were able to target patient prescriptions with a renal function below 30 mL/min. They are now analyzed on a daily basis. By the same method, prescriptions for people over 75 are also checked daily if they contain at least one PIMs among the most frequently found during the first phase of the audit. This new analysis method has been deployed in all of the establishment’s departments. It allows gaining relevance in patient targeting but does not increase the daily pharmaceutical analysis load.

Information and training meetings for prescribers and pharmacists were organized, during which the results of the first phase audit, the new pharmaceutical analysis method, and the prescription support documents were presented.

### 3.3. Second Audit Phase and Comparison

#### 3.3.1. Patient Characteristics

The population’s characteristics in the two phases did not show significant differences, except for the number of drugs prescribed per patient, which was higher in the second phase (Table 3).

#### 3.3.2. Evolution of Criteria between the Two Phases

No criterion considered compliant during the first phase became non-compliant for the second phase. The amount of non-compliant prescriptions decreased in the four remaining non-compliant criteria; this decrease was significant for illogical associations (*p* < 10^−2^), for clearance renal adaptation (*p* < 10^−3^) and for PIMs prescription (*p* < 10^−5^). Concerning the anticholinergic score, the decrease in non-compliant prescriptions was not significant, but the rate reached under 10% in the second phase. Only two criteria were non-compliant in the second phase: renal clearance and PIMs prescription remained over a 10% rate of non-compliance (Table 4). 

#### 3.3.3. Criterion 9: PIMs Prescription

The mean number of PIMs per patient was cut in half between the two audit phases, going from 0.81 PIM to 0.40 PIM per patient (*p* < 10^−3^; 133 PIMs for the first phase vs. 66 in the second phase). Despite a significant decrease in their prescription, PPIs remained the most prescribed PIMs (54.5%; *n* = 36; *p* = 0.02). Long half-life benzodiazepine (7.6%; *n* = 5; *p* = 0.86) and hydroxyzine (9.1%; *n* = 6; *p* = 0.79) prescriptions also decreased during the second phase, but not significantly.

#### 3.3.4. Criterion 8: Renal Clearance Adaptation

During the second phase, 10.3% of prescriptions were non-compliant regarding renal clearance adaptation (*n* = 17), with 19 drugs non-adapted to renal function. Every analgesic prescription was adapted for the second phase, but oral antidiabetic drugs represented 31.6% of non-adapted prescriptions (*n* = 6). The non-compliance rate neared 10% during the second audit phase.

#### 3.3.5. Criterion 6: Illogical Associations

The decrease of illogical associations was significant (*p* < 10^−2^). It was particularly important in therapeutic class redundancies (4.8% (*n* = 8) in the first phase vs. 0.6% (*n* = 1) in the second phase) and in combinations of more than three antihypertensive drugs (3.6% (*n* = 6) in the first phase vs. 1.2% (*n* = 2) in the second phase).

#### 3.3.6. Criterion 10: Overall Anti-Cholinergic Score

The mean anti-cholinergic score remained stable between phase 1 and phase 2, going from 1.73 (± 1.60) to 1.44 (±1.29) (*p* = 0.17); the number of prescriptions with an anti-cholinergic score >3 decreased from 12.1% to 7.3%, but this decrease was not significant (*p* = 0.19). With a non-compliance rate below 10%, this criterion became compliant during the second phase of the audit.

## 4. Discussion

The audit that we performed on the prescriptions for elderly patients in medical services mainly showed non-compliance for PIMs prescription, adaptation to kidney function, overall anti-cholinergic score, and illogical associations. The improvement actions undertaken have allowed significant enhancement of compliance for three criteria; and non-compliance rates for the illogical association and anti-cholinergic scores were under 10% after reevaluation.

Our study is the first to evaluate both the overall adaptation of the prescription to the elderly combined with a more specific analysis of PIMs prescribing in services of different specialties. This has provided a comprehensive view of the pharmaceutical management of the elderly in our institution.

The rate of non-adaptation of prescriptions to renal function found in our study is comparable to the literature. A study conducted exclusively in geriatric departments found 26% of prescriptions not adapted to renal function, whereas during our first phase nearly 25% of the prescriptions were not adapted [12]. After the implementation of improvement actions, approximately 10% of the prescriptions analyzed remained non-compliant, similar to the results found in another study conducted in elderly patients hospitalized in the cardiology department, which highlighted 14.4% of prescribed treatments not adapted to renal clearance [13]. The adaptation of all analgesic dosages during the second phase demonstrates the positive impact of the proposed improvement measures.

Regarding PIMs, the analysis of prescriptions by a pharmacist using tools such as the lists of Laroche [7], Beers [8], and the STOPP criteria of O’Mahony et al. [9] was a new approach in our institution. Many studies on prescribing have been carried out in different French hospitals, and the rates of prescriptions with PIMs ranged from 49.2% to 87.6% [13,14,15,16,17]. Our rates in the first phase were therefore in line with the literature, but the improvement actions enabled a considerable progression of compliance (57.6% vs. 32.1% after the improvement).

PPIs represent the most commonly prescribed PIMs in both phases, because either the indication or dosage was not justified. Some publications highlight the occurrence of adverse events that become more frequent with age: increased risk of fractures, Clostridium difficile-like digestive infections, pneumopathies, and vitamin deficiencies [18,19,20,21,22]. The number of non-compliant prescriptions is high, but it is important to note that this rate is lower than the rate found in the literature, which varies between 70 and 80% of non-compliant prescriptions [23,24,25].

Hydroxyzine and long half-life benzodiazepines were also among the most frequently prescribed PIMs, despite the reduction in prescriptions in the second round of the audit. Hydroxyzine is a molecule with high anti-cholinergic potency that causes sedation, urinary retention, constipation, and other adverse effects in the elderly [26]. It represents the most prescribed PIM in other studies; for example, its prescription rate reached 29.5% of the PIMs in a clinical audit conducted in geronto-psychiatry [16]. Long half-life benzodiazepines accumulate in the elderly and can cause confusion and daytime sedation, thus significantly increasing the risk of traumatic falls [26,27]. A study carried out in three different French cities on the elderly population showed a long half-life benzodiazepine impregnation rate of almost 10% [28]. The rates found in our audit are lower, and the newly implemented actions can only reinforce this gap.

The last class of PIMs targeted was central and peripheral alpha-blocking antihypertensive drugs. Alpha-blocking antihypertensive drugs induce a greater risk of orthostatic hypotension than other antihypertensive drugs [29]. Their prescription after reevaluation was halved (*p* = 0.89). Reevaluation of these treatments is hard for prescribers because blood pressure measurements must be taken outside of hospitalization and over several examinations in order to accurately assess blood pressure [30]. However, it has been possible, through the proposed actions, to reduce their prescription by limiting the number of instigations, and to encourage systematic screening for orthostatic hypotension when patients were to receive this therapeutic class.

The number of prescriptions containing an illogical association decreased significantly from 9.7% (*n* = 16) to 2.4% (*n* = 4) between the two phases (*p* = 0.009). Three types of illogical associations (IA) were highlighted in our study: therapeutic redundancies, the combination of a thiazide diuretic with an alpha blocker, and the prescription of more than three central antihypertensive drugs. Our results are comparable to those of studies conducted in retirement homes, long-stay units, and community pharmacies, which revealed rates between 3.5% and 8% of prescriptions with at least one illogical combination [31,32].

The decrease in the percentage of prescriptions with a high Anti-cholinergic Impregnation Coefficient (AIC) was not significant, illustrating the difficulty of routinely using the AIC score to limit prescriptions of anticholinergic drug combinations. The average anticholinergic score, over the totality of prescriptions between the two phases, remains significantly equivalent. The exchange with the prescribers during the presentation of the audit results revealed that the notion of the global atropinic score is not well known by the prescribers, and that the calculation is difficult to apply routinely.

Patient samples between the first and second audit phases were homogeneous and were therefore considered comparable. Only the number of drugs per prescription was significantly higher in the second audit round (9.5 vs. 10.3; *p* = 0.049) and could lead to a bias which, in our opinion, could only be in the direction of underestimating the improvement of compliance, as it could only lead to more inappropriate prescriptions in the second audit round.

During this clinical audit, no distinction was made between treatments initiated during hospitalization and treatments initiated in outpatient care and continued on admission. In this sense, we evaluated all the prescriptions and not only those initiated by the prescribers of our hospital center. Similarly, we were unable to measure the rate of inappropriate treatments stopped during hospitalization. This implies that the non-compliant prescriptions may have resulted either from non-reevaluation by hospital prescribers or from hospitalization. The various exchanges with prescribers concerning the audit reported difficulties surrounding the reassessment of prescriptions initiated in the community by the attending physician. Internists and geriatricians felt legitimate in re-evaluating treatments as a whole, unlike specialists for whom reassessing prescriptions for patients they do not usually follow is complicated. This is even truer when the length of hospitalization is short, and the treatment has been in place for a long time [33].

Our study had some limitations. First, the short observation period and the limited number of patients in a monocentric study may have limited the generalization of the results. Nonetheless, we assume the included patient to be representative of the source population given the inclusion of all patients in the recruiting units meeting the inclusion criteria in the study period. In addition, the lack of an a priori hypothesis supported by statistical analysis is also to be mentioned. Our results are first and foremost exploratory, and an intervention trial with a randomized group is needed to confirm the benefit of the implementation of improvement action. Such an approach has, nevertheless, allowed us to highlight the critical issues in elderly patient drug management in several medical specialties. In order to perform a similar study, it is important to involve as many services as possible with varied, but specific, evaluation criteria.

## 5. Conclusions

This targeted clinical audit made it possible to evaluate prescribing practices for the elderly in our institution’s medical department, and to reduce non-compliant practices through the implementation of improvement actions. The study revealed generally good prescribing practices from the first phase with six out of ten criteria in compliance, and significantly improved three out of four non-compliant criteria during the second phase of the audit.

At the end of the audit, potentially inappropriate drug prescriptions in the elderly and drugs not adapted to renal clearance were still present and required daily actions, through the analysis and re-evaluation of prescriptions with an active physician–pharmacist collaboration. The overall anti-cholinergic score of the prescriptions must be monitored closely, since this criterion has not been significantly improved. Our new method of targeting analyzed prescriptions, through the development of a daily alert on prescriptions of inappropriate drugs for the elderly and drugs not adapted to renal clearance, allows us to increase efficiency. Indeed, the pharmaceutical analysis time is not increased but allows more efficient targeting of at-risk patients. Combining this with the development of clinical pharmaceutical activities, through visits by the pharmacist to the patients in their rooms, can also promote the re-evaluation of prescriptions, increase the acceptance of pharmaceutical interventions, and thus lead to better-adapted prescriptions for the elderly. However, the use of a computerized clinical decision support system could help us increase efficiency and relevance [34]. These new tools are currently being studied to be adapted to our practice. A recent randomized clinical trial of the use of a computerized clinical decision support system in older adults showed an improvement in pharmacists’ abilities to make informed recommendations regarding anticholinergic and sedative medications, and to better align recommendations [35].

Finally, it is essential to continue to evaluate practices and regularly raise awareness among prescribers and pharmacists to maintain, or even improve, knowledge levels regarding the most optimal drug management.

## Figures and Tables

**Table 1:** Items selected for the evaluation grid of the clinical audit focused on prescriptions in the elderly person in medical service.

References	Items
CPGF/HAS	1. No more than two psychotropic drugs
	2. No more than one non-steroidal anti-inflammatory drug
	3. No more than one benzodiazepine or related drugs
	4. No cerebral vasodilator
	5. No contraindicated association between two drugs
	6. No illogical association
	7. Adaptation of the galenic formulation to the patient
	8. Adaptation of drugs to renal clearance
PIMs list	9. No Potentially Inappropriate Medications for the Elderly (PIMs)
AIC	10. Weak overall anti-cholinergic score (≤3)

**Table 2:** Non-compliance rates after completion of the first phase.

Criteria	Non-Compliance Rates % (*n*)
1. No more than two psychotropic drugs	5.5 (9)
2. No more than one non-steroidal anti-inflammatory drug	0.6 (1)
3. No more than one benzodiazepine or related drugs	3.6 (6)
4. No cerebral vasodilator	0 (0)
5. No contraindicated association between two drugs	0 (0)
6. No illogical association	9.7 (16)
7. Adaptation of the galenic formulation to the patient	1.8 (3)
8. Adaptation of drugs to renal clearance	24.9 (41)
9. No Potentially Inappropriate Medications for the Elderly (PIMs)	57.6 (95)
10. The total Anti-cholinergic score is week (≤3)	12.1 (20)

**Table 3:** Demographic and clinical data of patients included in the two audit phases.

	Phase 1	Phase 2	*p* Value
Number of patients	165	165	-
Sex ratio	1.04	0.67	*p* = 0.060
Men %(*n*)	50.9 (84)	40.0 (66)	-
Women %(*n*)	49.1(81)	60.0 (99)	-
Age (years)	84.2	83.9	*p* = 0.53
Length of stay (days)	9.9	9.8	*p* = 0.27
BMI (kg/m^2^)	26.4	25.4	*p* = 0.51
Renal function (ml/min/1.73 m^2^)	52.2	54.9	*p* = 0.41
Serum albumin level (g/L)	29.1	29.3	*p* = 0.59
Average number of drugs prescribed per patient	9.5	10.3	*p* = 0.05
Patients coming from home (*n*)	145	140	*p* = 0.52
Dependent elderly patients (*n*)	63	78	*p* = 0.12

**Table 4:** Criteria non-compliance rate comparison between the two phases.

Criteria	First Phases Non-Compliance Rates % (*n*)	Second Phases Non-Compliance Rates % (*n*)	*p* Value
1. No more than two psychotropic drugs	5.5 (9)	3.0 (5)	*p* = 0.41
2. No more than one non-steroidal anti-inflammatory drug	0.6 (1)	0 (0)	*p* = 1.00
3. No more than one benzodiazepine or related drugs	3.6 (6)	3.0 (5)	*p* = 1.00
4. No cerebral vasodilator	0 (0)	0 (0)	*p* = 1.00
5. No contraindicated association between two drugs	0 (0)	0 (0)	*p* = 1.00
6. No illogical association	9.7 (16)	2.4 (4)	*p* < 10^−3^
7. Adaptation of the galenic formulation to the patient	1.8 (3)	0 (0)	*p* = 0.25
8. Adaptation of drugs to renal clearance	24.9 (41)	10.3 (17)	*p* < 10^−3^
9. No Potentially Inappropriate Medications for the Elderly (PIMs)	57.6 (95)	32.1 (53)	*p* < 10^−3^
10. Weak overall anti-cholinergic score (≤3)	12.1 (20)	7.3 (12)	*p* = 0.19
